# Enabling and encouraging sleep deprivation among medical students

**DOI:** 10.36834/cmej.69918

**Published:** 2020-03-16

**Authors:** F. D’Eon Marcel

**Affiliations:** 1 University of Saskatchewan, Saskatchewan, Canada

We begin this editorial with a brief acknowledgement. Joanna Margaret Bates died January 18, 2020 after a long and prosperous career in family medicine and medical education with the University of British Columbia. She was a giant in her area, and few people in Canadian medical education were not touched by her scholarship. She will be truly missed.

I had been up very early to catch a flight to the US at 7:00 AM. The magazine in the seat pocket drew me in search for an easy Sudoku or crossword. I completed a couple of puzzles then uncharacteristically flipped through the rest of the pages. I was jolted awake by an article about sleep (and the lack, of which I have all too much personal experience).

Sleep is a personal and professional interest of mine in the context of medical student wellbeing and burnout. We know that poor sleep is common among medical students, but its prevalence is also higher than in non-medical students and the general population. Non-medical students and the general population are also sleep deprived which puts medical students in a class by themselves. If sleep quality and quantity are distributed among medical students along a somewhat normal curve, then about 15% of that population of medical students is at least one standard deviation worse than the mean. Since medical students are more sleep deprived than the general population, and 15% of them are much worse off, we should be alarmed.

Considerable evidence indicates that good quality sleep is important for cognitive and psychomotor performance and physical and mental health. Poor sleep has been implicated in burnout^1,2^and a host of other ill effects.^3^We need sleep, good sleep, and lots of it.^4^Not only are medical students more sleep deprived than the rest of us,^1,3^ those of us involved in medical education are complicit in an unspoken, unconscious conspiracy to cover up, minimize, and even boast about poor sleep habits and sleep deprivation. The World Health Organization has compared shift work, a prime driver of sleep deprivation, to first degree carcinogens like cigarette smoking.^4^How ironic, then, that those charged with improving the health of the population themselves have dysfunctional habits^1,5^ and attitudes about this most important health and hygiene practice. If I could put the health benefits of sleep into a bottle, I would make billions. We seem to ignore both the immense benefits of sleep and the disastrous consequences of its lack.

I have noticed (as I am sure most of you have) that medical students stay up late before exams (sometimes even pull an “all-nighter”). They manage to make it through their first call. Some brag about how little sleep they get. Many students and faculty joke about self-medicating (commonly called drinking coffee) to be able to manage the lack of sleep. Sleep deprivation does not train students to function better on less sleep. Sleep deprivation creates serious deficiencies.Whatwe get better at is complaining about it less, maybe. It seems few of us, if any, acknowledge or even attempt to address this serious public health crisis taking place right in our own medical schools.

While most medical schools tell their students that sleep is important and they should not skip or shorten their sleep, medical schools make it almost impossible for students to follow their advice. There is just too much to do and too much at stake to take the time to get a good amount of sleep. Often leaders in medical education play along or even lead the teasing and light humour about being overtired—a euphemism for sleep deprivation. All of this is readily observable, but the deeper, hidden message enables and encourages widespread sleep deprivation. This hidden curriculum seems to be more forcefulthan the official pleas to sleep well and prosper.

If we are serious about medical student mental and physical health, we need to open a conversation about sleep deprivation, our role in this situation, and how we can begin to find solutions that don’t blame our junior colleagues for their failure to care for themselves. This is a systemic issue and all of us need to be part of the answers. In some respects, I do hope that we “lose a little sleep” over this issue; it is that important.

This issue marks the first of the CMEJ in only its second decade, having begun publishing in 2010. We are proud of both the vision that leaders in medical education had for the CMEJ in Canada and the world, and the strong growth in numbers of submissions and pages published. We are especially pleasedwith the exponential growth in numbers of citations of articles published in the CMEJ (over 400 in 2019) and in the number of main page views (calculated to be over 100,000 in 2019). This phenomenal growth is due to the amount and quality of the articles published thanks to the spirit and hard work of our partners, supporters, volunteer editors, staff, and readers. The articles in this issue will help begin conversations about many other challenges facing medical education. We hope you enjoy and learn from them all.

Logan and her team in “Creating space for Indigenous healing practices in patient care plans” surveyed the Canadian Rheumatology Association membership to assess awareness and promote inclusion of Indigenous healing practices in patient care plans. Though the respondents were open to the idea of inclusion, they also had concerns. For Logan and team, this was an indication that there is a need for further learning and reconciliation in medicine.

Thoma and team in “Developing a dashboard to meet Competence Committee needs: a design-based research project” explained how CBME Competence Committees must effectively visualize learner assessment data and how dashboards can help. Using a design-based research process, they identified the data, analytics, and visualizations needed by Competence Committees, and then created the dashboard.

In “Does Emotional Intelligence at medical school admission predict future licensing examination performance?”, Wood and team looked at emotional intelligence (EI) scores as alternatives to traditional academic measures when selecting students for medical school. They studied whether there are correlations between EI scores and tests administered during admissions and medical school. Due to the low correlation between EI scores and licensure scores, they cautioned against using them as part of the admissions process.

Soleas and co-authors in their study “Developing Academic Advisors and Competence Committees members: A community approach to developing CBME faculty leaders” presented the competencies required for Academic Advisors (AA) and Competence Committee (CC) members in implementing competency-based medical education (CBME). They found that taking an active community-based approach to developing faculty leader competencies was valuable for transitioning to CBME. They hoped their findings would be a useful template for other institutions.

In “What knowledge is needed? Teaching undergraduate medical students to “go upstream” and advocate on social determinants of health”, Hayman et al. designed and piloted an intervention for medical students. They aimed to provide medical students the skills required to engage in policy change and empower them to be aware of and act on healthcare inequities. They advised teaching the next generation of physician advocates both practical advocacy skills and the relevant knowledge about health systems, health policy, and the SDOH.

Mador and co-authors in “Development of a novel conceptual framework for curriculum design in Canadian postgraduate trauma training” noted that a shift towards non-operative management of traumatic injuries and reduced resident work-hours have led to a decrease in trainees' surgical exposure to trauma. In this study, employing interview and focus groups, they did a needs assessment of trauma curricula for general surgery residents in Canada. They present a new conceptual framework to guide ongoing curricular reform for trauma care within the context of general surgery training with diminishing practice opportunities.

Cordovani and her team in “Maintenance of certification for practicing physicians: a review of current challenges and considerations” reviewedcurrent issues and challenges associated with MOC in medicine, including how to define medical competencies for practicing physicians, assessment, and how best to support physicians’ lifelong learning. In particular, they explored how the combination of self-monitoring, regular feedback, and peer support could improve self-assessment and could contribute to educationally valuable and clinically relevant ways of increasing physicians’ participation in MOC programs.

Tang and team in “Augmented reality in medical education: a systematic review” investigated, following PRISMA guidelines, the current state of augmented reality applications (ARAs) in medical education using publications from January 1, 2000 through June 18, 2018. They formulated an analytical model to assess the readiness of ARAs for implementation in medical education. Since the overall quality of the studies was poor, they could not yet recommend the adoption of ARAs into medical education.

In “Prevalence of intimidation, harassment, and discrimination among resident physicians: a systematic review and meta-analysis”, Bahji and Altomare summarized 52 cross-sectional studies in their meta-analysis. The overall pooled prevalence of intimidation, harassment, and discrimination was 64.1% (95% confidence interval [CI], 51.0-77.1). Verbal, physical, and sexual intimidation, harassment, and discrimination were the most common forms reported by residents. Training status (55.5%), gender (41.7%), and ethnicity (20.6%) were the most commonly identified risk factors. The most common sources of IHD were relatives/friends of patients, nurses, and patients. The prevalence of intimidation, harassment, and discrimination experienced by resident physicians continues to be high.

In “Eight ways to get a grip on implementing mindfulness sessions in medical schools,” Rac and Chakravarti outlined some practical pointers for getting a mindfulness program up and running in medical schools. They especially recommended an initial mandatory introductory session and then offering mindfulness practice sessions as one option from a suite of self-care practices.

In “Ten ways to get a grip on resident co-production within medical education change,” Dagnone and team drew on their extensive experience at Queen’s University with the implementation of CBME. They provided a guide for educators, learners, and institutions on how to leverage the interest and enthusiasm of trainees. They stressed empowering champions of change, building on early successes, and anticipating the change “dip”.

Persad, Verma, and Persad in “Endoscopy simulation for pre-clerkship students” report on a simulation session carried out with medical students during their gastroenterology block. Their goal was to demonstrate the clinical and anatomic implications of endoscopy, and to increase interest in gastroenterology. They report that their pre-clinical student group found the endoscopy simulation to be a useful addition to their gastroenterology studies.

Olszynski and co-authors in “The Clinical Ultrasonography Elective in Clerkship (CUSEC): A pilot elective for senior clerkship students at the University of Saskatchewan” created a clinical ultrasound (CUS) elective in clerkship. They aimed to give medical students the opportunity to put their CUS knowledge and skills into practice in real patient care. They found the medical students (1) met or exceeded their expectations in utilizing the CUS and (2) reported CUSEC to be a beneficial tool for patient care.

Ranpara’s commentary piece entitled “Designer babies” painted a picture of a future of genetic engineering where people create made-to-order designer babies: “People will be tall, handsome, and intelligent. They will be perfect. Who doesn’t want to be perfect?” Further in the future, when genetic modification still has flaws, people turned to artificial intelligence to create foolproof designer babies: “People will be tall, handsome, and intelligent. They will be perfect. Who doesn’t want to be perfect?”

In “A medical student goes to the country of Nunavut,” Thomsen D'Hont told the story of the challenges he faced when attempting to sign up for a clinical elective in the Canadian territory of Nunavut. He hoped that awareness of the barriers he faced will lead to changes in the portal system making it easier for medical students to spend credited time in the Canadian territory of Nunavut, rather than in the “foreign country” of Nunavut.

Finally, Rebecca Zhao provided a comic-style painting called “Flashback” to share her experience of recalling a traumatic incident. The graphic image intended to evoke discomfort so that viewers may more personally connect to her incident.

Enjoy!


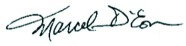
Marcel D’Eon, MEd, PhD Editor, CMEJ
